# Induction of robust immunity response in mice by dual-expression-system-based recombinant baculovirus expressing the capsid protein of porcine circovirus type 2

**DOI:** 10.1186/1743-422X-10-316

**Published:** 2013-10-28

**Authors:** Yu Ye, Xiaoliang Cheng, Jie Zhang, Tiezhu Tong, Wenyao Lin, Ming Liao, Huiying Fan

**Affiliations:** 1 Key Laboratory of Animal Vaccine Development, Ministry of Agriculture, Guangzhou 510642, China; 2 College of Veterinary Medicine, South China Agricultural University, Guangzhou 510642, China; 3 Huizhou Entry-Exit Inspection and Quarantine Bureau, Huizhou 516001, China

**Keywords:** Porcine circovirus type 2, Vaccine, Cap protein, Dual-expression-system, Immune response

## Abstract

**Background:**

Porcine circovirus type 2 (PCV2) is associated with post-weaning multisystemic wasting syndrome (PMWS), an emerging swine disease that causes progressive weight loss, dyspnea, tachypnea, anemia, jaundice, and diarrhea in piglets. Although *baculovirus* is an enveloped virus that infects insects in nature, it has emerged as a vaccine vector, and we used it to develop a novel candidate vaccine for a preventive or therapeutic strategy to control PCV2 infections.

**Methods:**

Immunoblotting analysis of recombinant baculovirus and immunofluorescent staining of baculovirus-infected cells were followed using anti-ORF2 monoclonal antibodies. The BALB/c mice were immunized intramuscularly with this baculovirus. The titers of antibodies were mensurated with a Cap-protein-specific enzyme-linked immunosorbent assay (ELISA) and a serum neutralization assay. The IFN-γ response in splenocytes harvested from immunized mice was measured by ELISA. Student's *t*-test was used to compare immune responses of different groups.

**Results:**

In this study, we successfully constructed a dual-expression-system-based recombinant baculovirus BV-GD-ORF2, which can display the PCV2 capsid (Cap) protein and VSV-G protein on the viral envelope and also expressing Cap protein on transduced mammalian cells, thereby functioning as both a subunit and a DNA vaccine. After infection, the Cap protein was expressed and displayed on the viral surface, as demonstrated with an indirect fluorescence assay and immunoblotting. The vaccination of mice with recombinant baculovirus BV-GD-ORF2 successfully induced robust Cap-protein-specific humoral and cellular immune responses.

**Conclusions:**

Our findings collectively demonstrate that the recombinant baculovirus BV-GD-ORF2 is a potential vaccine against PCV2 infections.

## Background

Porcine circovirus type 2 (PCV2), a member of the family *Circoviridae*, is recognized as the causative agent of the various clinical manifestations of PCV-associated diseases (PCVAD), although other factors influence the severity of the clinical symptoms [1]. To combat the growing problems associated with PCV2-associated disease, several commercial PCV2 vaccines have been developed and been shown to provide some degree of protection against PCV2 infection [2,3]. In the present study, a new approach to the prevention and control PCV2 was explored using a baculovirus dual expression system to express the capsid (Cap) protein of PCV2 as a subunit vaccine and a DNA vaccine.

The baculovirus *Autographa californica* multiple nuclear polyhedrosis virus (AcMNPV) has been used traditionally as an excellent tool to overexpress recombinant proteins in insect cells [4-6]. Because the baculovirus can enter mammalian cells and mediate the expression of transgenes under a promoter that is active in mammalian cells [7], baculoviral vectors have been exploited as versatile vaccine vehicles to produce candidate vaccines against various pathogens [8-10]. It has also been reported that a pseudotype baculovirus displaying the glycoprotein of vesicular stomatitis virus (VSV-G) on its envelope can extend the host range of the baculovirus and enhance its resistance to inactivation by animal serum complement [11-13].

Recently, AcMNPV has been further engineered for use as a new eukaryotic display system to express exogenous peptides on the surface of the viral envelope. This display strategy relies on thegp64 protein, which is the major envelope protein of the baculoviruses, which has an amino-terminal signal peptide (SP), a mature transmembrane domain (TM) and a cytoplasmic tail domain (CTD). For the surface display of exogenous peptides, a heterologous peptide was inserted between the SP and mature domain of gp64. After its expression with the native gp64, the fusion protein is translocated to the plasma membrane and incorporated into the baculoviral envelope. This method has been extended to develop pseudotyped baculoviruses as a potential vaccine delivery platform. Several research groups have demonstrated that direct vaccination with pseudotyped baculoviruses can induce high titers of antigen-specific antibodies [14-16].

Based on the characteristics of the baculoviruses as a gene delivery system and surface display system together, a dual-expression-system-based recombinant baculovirus BV-GD-ORF2 was constructed in this study, which can display the PCV2 Cap protein and the VSV-G protein on the viral envelope and expresses the Cap protein when it is used to transduce mammalian cells. The first objective of this study was to effectively display the functional Cap protein on the baculoviral envelope, hoping that the Cap protein would retain its superior immunogenicity after in vivo immunization. Our second objective was to efficiently express the Cap protein in transduced mammalian cells. The third objective was to display the VSV-G protein on the viral envelope to boost the baculovirus resistance to complement. The potential utility of BV-GD-ORF2 as a vaccine was evaluated in a mouse model. Robust humoral and cellular immune responses were successfully induced in mice immunized with the recombinant baculovirus BV-GD-ORF2. These results suggest that a PCV2 vaccine based on the baculovirus dual expression system can be used as an alternative strategy to protect against PCV2 infection. To our knowledge, this is the first study to establish a PCV2 vaccine based on the baculovirus dual expression system.

## Results

### Construction of the baculovirus dual expression system

**Figure 1:**
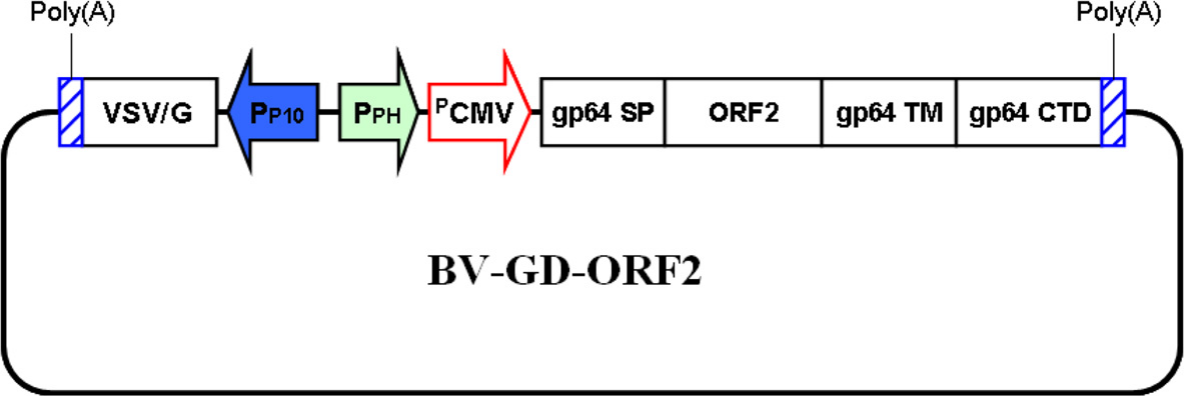
**Schematic representation of the structure ofBV-GD-ORF2.** The ORF2 gene cassette consists of the gp64 signal sequence (SP), the ORF2 gene (ORF214–234) fused to the N-terminus of the AcMNPV major envelope protein gp64 gene (gp6425–517), and the poly(A) sequence, which was driven by a dual promoter containing the CMV immediate-early enhancer/promoter (pCMV-IE) and the polyhedrin promoter (PPH). The VSV-G expression cassette was driven by the p10 promoter (PP10). ORF214–234:ORF2 corresponding to amino acids 14–234, but lacking the N-terminal nuclear localization signal peptide of the capsid protein of PCV2. Gp6425–517: gp64 corresponding to amino acids 25–517.VSV/G: the glycoprotein of vesicular stomatitis virus.

**Figure 2:**
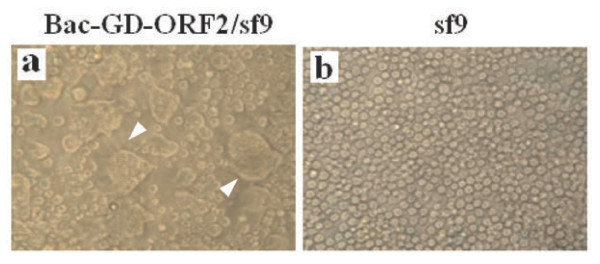
**Characterization of BV-GD-ORF2-infected Sf9 cells.** Syncytium formation in Sf9 cells infected with BV-GD-ORF2 as indicated by the white arrow **(a)**, but not in the mock-infected Sf9 cells **(b)**. The images were captured at 72 h posttransfection. Original magnification, ×100.

The recombinant baculovirus BV-GD-ORF2 was constructed as described in the Methods (Figure 1). The infection of Sf9 cells with BV-GD-ORF2 caused in extensive cell-cell fusion (Figure 2). This phenotype is attributable to the very high expression level of VSV-G protein, which has membrane-fusion activity, under the control of the polyhedrin promoter (PPH).

**Figure 3:**
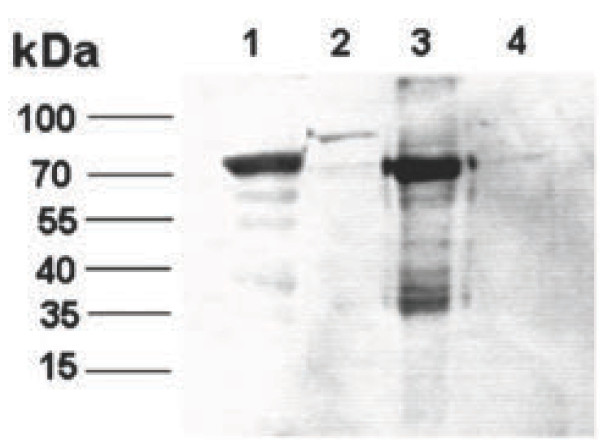
**Immunoblotting analysis of recombinant AcMNPV particles using anti-ORF2 monoclonal antibodies.** Sf9 cells infected with BV-GD-ORF2 (lanes 1); Sf9cells infected with AcMNPV-WT (lanes 2); complete BV-GD-ORF2 virions (lanes 3); and purified nucleocapsids from BV-GD-ORF2 virions treated with Triton X-100 (lanes 4). All were treated with lysis buffer and subjected toimmunoblotting. Expression of the ORF2–gp64fusion protein was examined.

To investigate whether the Cap protein was displayed on the membrane of BV-GD-ORF2, the purified viral particles were analyzed with immunoblotting with an anti-ORF2 monoclonal antibody. A protein of an approximately 75 kDa protein (of the predicted size) was presented in the lanes loaded with the recombinant baculovirus BV-GD-ORF2 or with BV-GD-ORF2-infected Sf9 cells, but not in lanes containing pellets of viral nucleocapsids only or in those lanes loaded with AcMNPV wild-type (AcMNPV-WT) infected Sf9 cells (Figure 3). Our observations indicated that the presence of both VSV-G and PCV2 Cap protein does not significantly reduce the displaying efficiency of Cap protein on baculovirus membrane.

**Figure 4:**
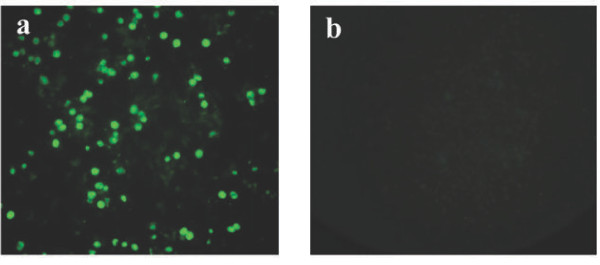
**Immunofluorescent staining of BV-GD-ORF2-infected cells.** PK-15 cells were transfected with BV-GD-ORF2 **(a)** or AcMNPV-WT **(b)** at an MOI of 10, respectively. At 48 h posttransfection, the cells were fixed with absolute methanol, and processed for an indirect immunofluorescence assay with mouse monoclonal antibody directed against ORF2. The bound antibodies were detected with FITC-labeled anti-mouse IgG and observed with fluorescence microscopy (green). Original magnification, ×100.

To test the ability of BV-GD-ORF2 to express the Cap protein in mammalian cells, PK-15 cells were transduced with BV-GD-ORF2 and AcMNPV-WT. Distinct fluorescent signals were detected in the BV-GD-ORF2-transduced cells but not in the cells transduced with AcMNPV-WT (Figure 4). These results demonstrated that BV-GD-ORF2 not only displays the PCV2 Cap protein on the viral envelope but also expresses it in mammalian cells.

### Humoral immune responses in mice immunized with BV-GD-ORF2

To determine whether BV-GD-ORF2 can induce PCV2-specific immune responses in vivo after direct immunization, BALB/c mice were immunized intramuscularly with BV-GD-ORF2, pc-ORF2, AcMNPV-WT or phosphate-buffered saline (PBS). Cap-protein-specific antibodies and neutralizing antibodies were determined 3, 6, and 9 weeks after the primary immunization.

**Figure 5:**
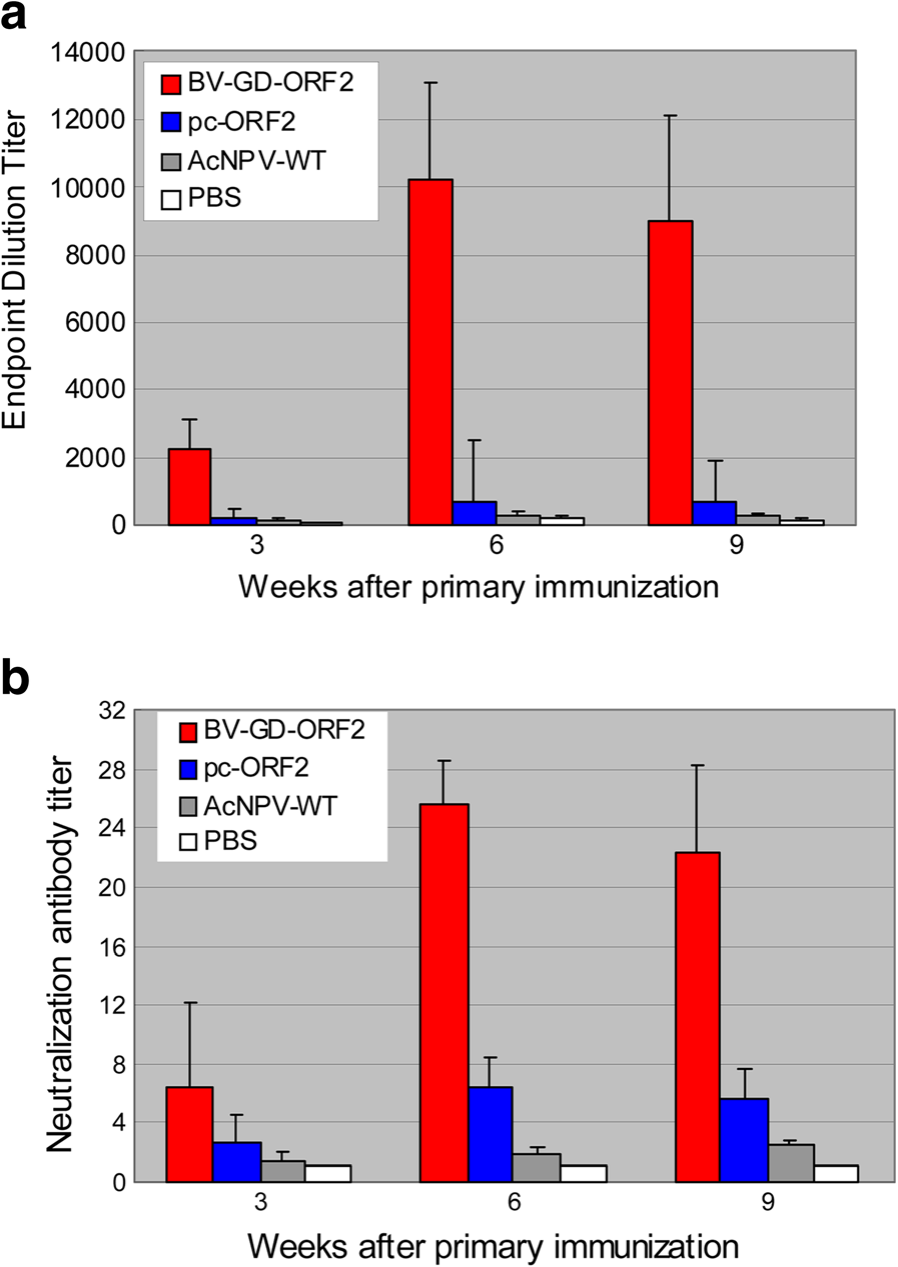
**Antibody responses in mice immunized with of BV-GD-ORF2.** Mice were immunized intramuscularly with BV-GD-ORF, pc-ORF2, AcMNPV-WT or PBS. An identical booster immunization was given 3 weeks later. Serum was obtained at 3, 6, and 9 weeks. Antibody levels were analyzed with a Cap-protein-specific ELISA **(a)** and a serum neutralization assay **(b)**. All data are means ± SD.

As shown in Figure 5a, 3 weeks after the primary immunization, Cap-specific antibodies were detected in all the mice immunized with BV-GD-ORF2 or pc-ORF2. Following the booster immunization, the mean antibody titers increased rapidly in the mice treated with BV-Dual-ORF2 or pc-ORF2, and there was a significant difference between the two groups at 6 weeks (P < 0.01) and 9 weeks (P < 0.01) after the primary immunization.

Serum samples were evaluated for their capacity to neutralize PCV2 strain GD-68 in a serum neutralization assays. As shown in Figure 5b, during the experimental period, mice immunized with the BV-GD-ORF2 developed significantly higher PCV2-specific neutralizing antibody titers than mice injected with pc-ORF2 (P <0.01), and the average neutralizing antibody titers in the mice vaccinated with BV-GD-ORF2 was up to 1:25.6 at 6 weeks after the primary immunization. As expected, none of the negative control mice treated with AcMNPV-WT or PBS produced detectable Cap-protein-specific antibodies or neutralizing antibodies throughout the whole experimental period.

### Cellular immune response in mice immunized with BV-GD-ORF2

**Figure 6:**
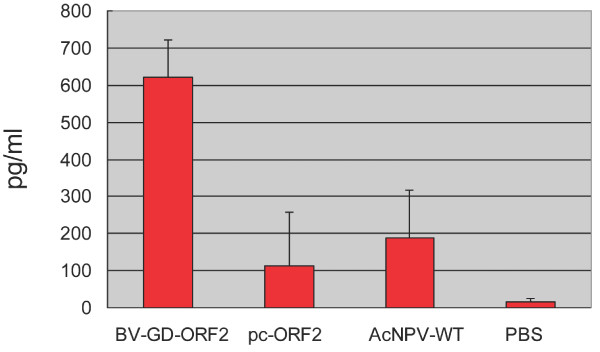
**The IFN-γ response in splenocytes harvested from immunized mice was measured by ELISA.** Mice were immunized as in Figure 5. Splenocytes were isolated at 6 weeks after the primary immunization and stimulated with or without purified Cap protein in vitro for a defined time. The IFN-γ levels in the supernatant were analyzed with an ELISA. Data are means ± SD.

To characterize the cell-mediated immune responses in mice immunized with BV-GD-ORF2, splenocytes were isolated from the immunized mice at 6 weeks after the primary immunization and interferon γ (IFN-γ) production in splenocytes restimulated with the recombinant Cap protein was measured with an ELISA. As shown in Figure 6, mean IFN-γ levels of 621 pg/ml or 112 pg/ml were detected in mice inoculated with 1× 10^9^ PFU of BV-GD-ORF2 or pc-ORF2, respectively, and the difference between the two groups was significant (P < 0.01). Splenocytes from mice immunized with AcMNPV-WT also showed a relatively high nonspecific IFN-γ response (189 pg/ml).

Because nearly all swine herds are seropositive for PCV2 and there are insufficient numbers of specific-pathogen-free swine, and because experimentation with swine is expensive, our results were obtained with animals other than swine. Therefore, future studies should include data from swine if we are to completely understand the potential utility of candidate PCV2 vaccines based on the dual expression system of recombinant baculoviruses displaying immunogens.

## Discussion

Porcine circovirus 2 is the principal infectious agent implicated in the development of PMWS [17,18] and has also been associated with many other conditions such as porcine dermatitis and nephropathy syndrome (PDNS) and congenital tremors (CT), which are now collectively known as PCVAD [19]. Despite the use of preventive measures involving swine housing and herd management, high PCV2 seroprevalence and PMWS are found in many herds. The prevention of this disease maybe possible if vaccination-induced immunity can be developed in piglets before the time at which waning maternal immunity makes the animals susceptible to PCV2 infection [20]. Although several commercial vaccines have been developed to control PCV2 infections, there are potential safety problems associated with killed vaccines, including their incomplete inactivation and an increased risk of allergic reactions arising from the large amounts of antigen involved. Less expensive vaccines will make vaccination far more available for livestock, whereas genetically engineered vaccines, such as baculovirus-based vaccine, especially for the farms in developed countries. Thus, the development of an effective and affordable vaccine against PCV2 infection is an acceptable strategy for the prophylaxis of PMWS.

In this study, the dual-expression-system-based recombinant baculovirus BV-GD-ORF2 has been developed, which possesses two gene cassettes: PP10 promoter-controlled VSV-G expression cassette and CMV immediate-early enhancer (CMV-IE) and PPH promoters-controlled PCV2 ORF2 expression cassette. We investigated the efficacy of BV-GD-ORF2 as a vaccine against PCV2. Our results clearly showed that immunization with BV-GD-ORF2induced robust humoral and cellular immune responses in a mouse model, indicating that BV-GD-ORF2 has value as a candidate vaccine to prevent and control PCV2.

Previous studies have shown that the PCV2-neutralizing antibodies were correlated, to some degree, with the efficacy of protection against PCV2. In this study, mice immunized with BV-GD-ORF2 developed significantly higher PCV2-specific neutralizing antibody titers than did those immunized with pc-ORF2, probably because of the nature of the vaccine form. BV-GD-ORF2 is capable of the surface-display of the PCV2 Cap protein on the viral envelope and expresses it on transduced mammalian cells, so it plays dual roles as a subunit vaccine and a DNA vaccine, whereas pc-ORF2 only expresses Cap protein and functions as a DNA vaccine. In contrast, VSVG is displayed on the envelope of the pseudotyped baculovirus. The VSVG-modified virus not only prevents attack by the complement system in the mammalian serum, but also the efficiency of gene transfer into mammalian cells in vivo [21]. The Cap protein displayed on the envelope of BV-GD-ORF2 allows it to engage antigen-presenting cells (APCs) and activate the ORF2-specific immune reactions via the major histocompatibility II (MHC-II)-mediated antigen-presentation pathway, thus leading to more potent immune responses [22]. Moreover, unlike the DNA vaccine, the baculovirus can directly transduce the APCs [23,24] resident in the muscle tissues and thus present antigen more efficiently to dendritic cells (DCs), which are the most important APCs [25].

The protective immunity elicited by PCV2 vaccines is considered to be primarily mediated by the humoral response, although few data are available that describe the role of cell-mediated immunity in protection against PCV2. Previous studies have suggested that high levels of IFN-γ have a deleterious effect on protective immunity [26]. However, Shen et al. recently reported that protective immunity against PCV2 in mice was mediated by Cap-specific CD8^+^ cells and a seroneutralization response [27]. Fort et al. also demonstrated that PCV2-specific cellular responses in pigs contributed, together with neutralizing antibodies, to viral clearance [2]. A previous study also showed that immunization with adjuvant-free baculovirus displaying the rodent malaria *Plasmodium berghei* circumsporozoite protein on its envelope induced high levels of antibodies and IFN-γ-secreting cells, and protected 60% of mice against sporozoite challenge [28]. In this study, we observed a significantly enhanced cell-mediated immune response in mice immunized with BV-GD-ORF2 compared with that of mice immunized with pc-ORF2. A higher background of nonspecific IFN-γ production in splenocytes harvested from the baculovirus-injected mice was also observed. This might be because gp64, the baculovirus envelope protein, recognizes the TLR9 molecule and thus activates the innate immune response [29]. This is consistent with the results of a previous study in which intranasal immunization with WT baculovirus alone provided sufficient protection from lethal challenge with the H1N1 influenza virus [8].

At present, there are at least four commercially available PCV2 vaccines, including subunit vaccines that express the Cap protein from baculovirus, inactivated PCV2, and a PCV1/PCV2 chimeric vaccine [27]. However, the propagation of PCV2 or the production of a recombinant Cap protein in expression systems designed for vaccine production is a time-consuming, expensive and laborious process [30]. In contrast, the baculovirus dual expression system allows the rapid generation of recombinant baculoviruses, and high titers (>10^9 ^PFU/ml) can be produced easily. In particular, the inability of baculoviruses to replicate in mammalian cells makes them attractive candidate vectors for transgenic expression studies if they are engineered to contain suitable expression cassettes [31]. Considering the safety, the cost-effectiveness, and the simple scale-up required to produce high-titer recombinant baculoviruses, the baculovirus dual expression system is a promising method of vaccine development, superior to other viral systems.

The baculovirus vector also has a large genome, which allows the insertion of large foreign DNA fragments or the construction of multivalent vaccines possible. In a further study, we plan to improve the immunization efficiency of this system by the tandem expression of multiple Cap proteins to produce multivalent vaccines, and display a single-chain antibody fragment specific for some pathogens or ligand-directed specific targeting of baculovirus to increase the tropism in virus-infected mammalian cells. We are also ready to co-express the Cap protein and other immunogenic proteins of some important pathogens, such as the GP5 and M proteins of porcine reproductive and respiratory syndrome virus (PRRSV), in order to develop a potential divalent candidate vaccine against PRRSV and PCV2 infections.

In summary, this study demonstrates that the vaccine BV-GD-ORF2, based on the baculovirus dual expression system, can be used as an alternative strategy to prevent and control PCV2. Although mice were used as the experimental animal model in which to evaluate the immunogenic efficacy of the PCV2 vaccine [32,33], other experiments are required to determine the immunogenicity and protective effect of this recombinant baculovirus in pigs [34].

## Conclusions

A dual-expression-system-based recombinant baculovirus BV-GD-ORF2 displays the PCV2 Cap protein with the baculovirus envelope protein gp64 TM and CTD on the viral envelope and also expresses it on transduced mammalian cells, thereby functioning as both a subunit vaccine and a DNA vaccine. The vaccination of mice with BV-GD-ORF2 successfully induced robust Cap-protein-specific humoral and cellular responses. Collectively, our results indicate that the recombinant baculovirus BV-GD-ORF2 is a potential vaccine against PCV2 infection.

## Methods

### Cell lines

The porcine kidney cell line (PK-15) free of PCV1 contamination (ATCC CCL 33) was maintained in Dulbecco’s modified Eagle’s medium (DMEM, Invitrogen, Carlsbad, CA, USA), supplemented with 10% (v/v) heat-inactivated fetal bovine serum (FBS, Invitrogen), 100 g/mL of streptomycin and 100 IU/mL of penicillin. *Spodoptera frugiperda* cells (Sf9) were used to propagate the WT and recombinant baculoviruses and were cultured in Grace’s insect media (Invitrogen) supplemented with 10% heat-inactivated FBS at 27°C. PCV2 strain GD-68 used in this study was originally isolated from a pig with naturally occurring PMWS.

### Construction of recombinant baculovirus

To generate the recombinant baculovirus BV-GD-ORF2, VSV–G cDNA with the β–globin terminator was PCR amplified from pVSV–G (Clontech, Mountain View, CA, USA) and subcloned into the shuttle vector pFastBac™Dual plasmid (Invitrogen). The ORF2 gene lacking the N–terminal nuclear localization signal peptide was amplified from PCV2 genomic DNA by PCR and inserted between the sequences encoding the gp64 signal peptide and the transmembrane domain of pBACsurf–1 vector (Novagen, Madison, WI, USA). To construct pCMV–surf–ORF2, a 2.1-kb fragment of the surf–ORF2 fusion gene was excised from psurf–ORF2 by digestion with *Nhe*I and *Hind*III, and inserted into the *Nhe*I/*Hind*III sites in the pcDNA3.1(+) vector (Invitrogen, Carlsbad, CA). A cassette comprised of CMV-IE promoter and the surf-ORF2 fusion gene was amplified by PCR and subcloned into plasmid pFastBac™ Dual after *Sal*I/*Hind*III treatment to generate pBac-GD-ORF2. The resultant pBac-GD-ORF2 plasmid thus contained the PCV2 ORF2 gene driven by the CMV-IE + PPH promoters and the VSV–G gene driven by the PP10 promoter.

All procedures for the production of recombinant baculovirus BV-GD-ORF2 were performed according to the manufacturer's manual of Bac-to-Bac® Baculovirus Expression System (Invitrogen). Viral particles were purified with two rounds of sucrose gradient ultracentrifugation following standard protocols [35], and infectious titers were determined with the BD BacPAK™ Baculovirus Rapid Titer Kit (Clontech).

### Immunoblotting analysis

To investigate whether ORF2 is displayed on the envelop of BV-GD-ORF2, the purified virions were treated with 1% Triton X-100 to disrupt the virion structure, and then the viral nucleocapsids were collected by ultracentrifugation. The viral nucleocapsids, purified recombinant baculovirus and infected cell lysates in PBS (pH 7.4) were then mixed with lysis buffer (50 mM Tris–HCl [pH 6.8], 0.1 M dithiothreitol, 2% sodium dodecyl sulfate [SDS], and 10% glycerol) and resolved by SDS-12% polyacrylamide gel electrophoresis. Fractions containing the purified baculovirus were then transferred onto nitrocellulose membrane. Immunoblotting was performed as previously described [9] with an anti-ORF2 monoclonal antibody (Ingenasa Inc., Spain).

### Immunofluorescence assay

PK-15 cells were seeded at a concentration of 2.5 × 10^5^ cells/well into six-well tissue culture plates (Nunc, Rochester, NJ, USA) and transduced with purified baculovirus particles at a multiplicity of infection (MOI) of 10. After incubation for 48 h, the cells were fixed for 15 min with absolute methanol (100%) and incubated with anti-ORF2 monoclonal antibody (Ingenasa Inc.), and then with fluorescein-isothiocyanate (FITC)-conjugated rabbit anti-porcine IgG (Sigma, St. Louis, MO, USA). Fluorescent images were examined under an inverted fluorescence microscope (Olympus IX70, Japan).

### Mouse immunization schedule

Six-week-old female BALB/c mice were purchased from Southern Medicine University, Guangzhou, China, and cared for according to the Chinese Guidelines for Laboratory Animals in the microisolator units at Southern China Agricultural University. Three groups of mice (eight per group) were vaccinated intramuscularly twice at 3-week intervals with 10^9^ PFU of BV-GD-ORF2, 10^9^ PFU of AcMNPV wild-type (AcMNPV-WT) or 100 μg of pc-ORF2.A fourth group was injected with 100 μl of PBS and used as the control group. Serum samples were collected 3, 6, and 9 weeks after the primary inoculation to detect the Cap protein-specific ELISA antibodies and neutralizing antibodies against PCV2. Six weeks after the primary immunization, three mice from each group were killed for a cell-mediated immune assay.

### Serological testing

An endpoint ELISA and serum neutralization assay were performed as previously described [9].

### IFN-γ release assays

Mouse splenocytes were prepared as previously described (Fan et al., 2008), and incubated in 24-well flat-bottomed plates (1 × 10^6^ cells/well) in the presence of Cap protein (20 μg/ml). After incubation for 72 h, the culture supernatant was harvested and the presence of IFN-γ was measured with a commercially available mouse IFN-γ ELISA kit (Biosourse, USA) according to the manufacturer's instructions. The concentrations of mouse IFN-γ in the samples were determined from a standard curve.

### Statistical analysis

Student's *t*-test was used to compare the humoral and cell-mediated immune responses of the different groups. P values of <0.05 were considered statistically significant.

## Competing interests

None of the authors has any financial or personal relationships that could inappropriately influence or bias the content of the paper.

## Authors' contributions

XLC, ML and HYF conceived the study. XLC, YY, JZ, WYL, TZT and HYF designed and performed experiments. XLC, YY and HYF analyzed the data. YY and HYF wrote the manuscript. All authors read and approved the final manuscript.
